# Novel Immunotherapeutic Approaches for Head and Neck Squamous Cell Carcinoma

**DOI:** 10.3390/cancers8100087

**Published:** 2016-09-22

**Authors:** Darrin V. Bann, Daniel G. Deschler, Neerav Goyal

**Affiliations:** 1 Division of Otolaryngology, Head and Neck Surgery, Department of Surgery, College of Medicine, The Pennsylvania State University, 500 University Drive, P.O. Box 850, Hershey, PA 17033, USA; dbann@hmc.psu.edu; 2 Institute for Personalized Medicine, College of Medicine, The Pennsylvania State University, Hershey, PA 17033, USA; 3 Department of Otolaryngology, Harvard Medical School, Massachusetts Eye and Ear Infirmary, Boston, MA 02114, USA; daniel_deschler@meei.harvard.edu

**Keywords:** head and neck squamous cell carcinoma, immunotherapy, tumor vaccine, monoclonal antibody, oncolytic viruses, immune checkpoint inhibitors

## Abstract

The immune system plays a key role in preventing tumor formation by recognizing and destroying malignant cells. For over a century, researchers have attempted to harness the immune response as a cancer treatment, although this approach has only recently achieved clinical success. Head and neck squamous cell carcinoma (HNSCC) is the sixth most common cancer worldwide and is associated with cigarette smoking, alcohol consumption, betel nut use, and human papillomavirus infection. Unfortunately, worldwide mortality from HNSCC remains high, partially due to limits on therapy secondary to the significant morbidity associated with current treatments. Therefore, immunotherapeutic approaches to HNSCC treatment are attractive for their potential to reduce morbidity while improving survival. However, the application of immunotherapies to this disease has been challenging because HNSCC is profoundly immunosuppressive, resulting in decreased absolute lymphocyte counts, impaired natural killer cell function, reduced antigen-presenting cell function, and a tumor-permissive cytokine profile. Despite these challenges, numerous clinical trials testing the safety and efficacy of immunotherapeutic approaches to HNSCC treatment are currently underway, many of which have produced promising results. This review will summarize immunotherapeutic approaches to HNSCC that are currently undergoing clinical trials.

## 1. Introduction

Over the last several decades it has become increasingly clear that the immune system plays an important role in cancer prevention through a process called immune surveillance [1]. In turn, the selective pressure exerted by the immune system causes tumors to develop multiple mechanisms to escape immune detection, including the induction of immune tolerance, local suppression of the immune response, and systematic disruption of T-cell signaling [2]. Recently, there has been intense focus on immunotherapy, which the National Cancer Institute defines as “biologic therapy that uses substances to stimulate or suppress the immune system to help the body fight cancer, infection, and other diseases”. However, the idea that the immune system can be used to treat or cure cancer is not new; in fact, the first studies using immunomodulation for cancer therapy were conducted over a century ago [3]. Despite this long history, modern cancer immunotherapies including monoclonal antibodies, tumor vaccines, oncolytic viruses, and proinflammatory molecules, have only met significant clinical success over the last decade [4].

One cancer that may benefit from immunotherapy is head and neck squamous cell carcinoma (HNSCC). Worldwide, HNSCC represents the sixth most common cancer, resulting in approximately 550,000 diagnoses and 300,000 deaths per year [5]. The relatively poor overall survival (OS) for HNSCC patients despite advances in surgical techniques, chemotherapy, and radiation therapy results, in part, from an inability to further intensify therapy due to unacceptable morbidity, and partly from the profound immunosuppressive effects of the tumor itself [6]. Therefore, significant efforts have been directed towards stimulating the immune response against HNSCC to improve survival and reduce morbidity associated with the disease. In this review, we outline immunomodulatory approaches to HNSCC treatment that are currently under investigation as Phase I, II, or III clinical trials.

## 2. Identification of Clinical Trials

Current clinical trials for HNSCC immunotherapies were identified by querying the ClinicalTrials.gov database for “HNSCC” + “immune” or “squamous cell carcinoma” + “head and neck” + “immunotherapy”. At the time of the search in April 2016, this approach yielded 53 trials, which were manually reviewed for inclusion. To focus on current clinical trials, studies that were withdrawn without results were excluded, while trials that were terminated or completed without posted results were included. In total, 33 trials covering 22 different immunotherapeutic approaches were retained for discussion (Table 1). For clarity, the immunotherapeutic agents are divided into four broad categories based on the class of therapy, with further sub-classification of different agents based on the actual or proposed mechanism of action (Table 1). The four major classes of immunotherapy covered by this review are vaccine therapies, monoclonal antibodies, oncolytic viruses and active immunotherapeutics, and immunomodulators (Table 1).

## 3. Vaccine Therapy

Anticancer vaccine therapies involve generating an antitumor immune response by presenting a tumor-associated antigen (TAA) plus an immunostimulatory adjuvant, resulting in immune sensitization to tumor antigens. To date, several vaccination strategies have been applied, including the transfection of TAA expression plasmids into patient tissues (DNA vaccines), the administration of TAA peptides (peptide vaccines), and the use of cultured human or microbial cells to generate an antitumor immune response.

### 3.1. DNA Vaccines

#### 3.1.1. INO-3112

INO-3112 is a combination of two previously developed DNA vaccines, VGX-3100 and INO-9012, originally developed for the treatment of cervical cancer. The expression plasmids contained within the vaccine produce E6 and E7 proteins from human papillomavirus (HPV)16 and HPV18, respectively, resulting in an HPV-specific CD8+ T cell response. Given the selectivity for HPV proteins, this vaccine is only appropriate for HPV-positive HNSCC. The vaccine is administered as an intramuscular injection of plasmid DNA once every three weeks to a total of four doses. Because the plasmid-encoded antigens must be expressed to generate an immune response, each injection is accompanied by electroporation with the CELLECTRA^®^ device, which causes surrounding myocytes to incorporate and express the vaccine plasmids. Interim results from Phase I trials of INO-3112 including 19 patients showed that 80% (4/5) of patients tested had elevated anti-E6/E7 antibody titers for HPV16 and HPV18 and that 87.5% (7/8) of patients tested demonstrated elevated CD8+ T cell responses following vaccination [7,8].

Adverse effects in the study group were generally mild and included injection site pain (58%), local erythema (21%), hematoma (13%), and swelling (13%). All adverse events were Grade 2 or lower. Efficacy endpoints have yet to be reported for INO-3112, however a phase II trial of VGX-3100, which is included in INO-3112, demonstrated similar immune responses and resulted in regression of cervical intraepithelial neoplasia grade II or III lesions in 48.2% of patients in the experimental group compared to disease regression in only 30% of patients receiving placebo [9]. INO-3112 is currently being tested in a Phase I/II trial for HNSCC with results expected in 2017.

#### 3.1.2. Allovectin-7

The Allovectin-7 vaccine is a DNA/lipid complex containing plasmids encoding the Human Leukocyte Antigen-B7 (HLA-B7) heavy chain and β_2_ microglobulin, resulting in the expression of the complete major histocompatibility complex type-I (MHC-I) molecules on the surface of tumor cells. Many tumor types evade immune recognition by reducing MHC-I expression, thereby preventing the presentation of tumor antigens on the cell surface [10,11]. Accordingly, increasing MHC-I may facilitate immune recognition of tumor antigens as foreign, promoting antitumor immunity. In addition, because the HLA-B7 molecule expressed by Allovectin-7 is frequently allogenic to the recipient, expression of HLA-B7 itself may promote a strong inflammatory response. In contrast to INO-3112, which requires electroporation for plasmid expression, the DNA/lipid complex used by Allovectin-7 allows for direct uptake of plasmid DNA by the myocytes surrounding the injection site. Phase I and II trials of Allovectin-7 in HNSCC patients demonstrated that the vaccine is safe and well-tolerated, with 33% of patients exhibiting partial or complete tumor response [12]. However, no HLA-B7-positive patients demonstrated a clinical response to the vaccine therapy, suggesting that the antitumor effects of the vaccine may be due to the immune response to HLA-B7 itself, and not tumor antigens [12]. Additionally, a phase III trial of Allovectin-7 in metastatic malignant melanoma failed to reach its primary or secondary endpoints of tumor response and improved OS compared to chemotherapy [13], which resulted in the termination of the Allovectin program in 2013. At the time, a Phase II/III trial for the use of Allovectin-7 in HNSCC was underway, although no results were reported before the program was cancelled.

### 3.2. Peptide Vaccines

#### 3.2.1. MAGE-A3/HPV16

The MAGE-A3 vaccine was originally developed for treatment of non-small cell lung carcinoma, although a phase III trial of >2000 patients failed to demonstrate efficacy over placebo for this patient population [14]. The vaccine targets MAGE-A3, a human protein that is highly expressed in a variety of tumors and during embryogenesis, but is only found in the placenta and testis of adults [15]. The MAGE-A3/HPV16 combines MAGE-A3 peptides with furin-sensitive linkers and the GL-0810 vaccine containing an HPV16-specific peptide. Importantly, peptides from both vaccines contain the human immunodeficiency virus-1 (HIV-1) Tat membrane translocation sequence, which allows the peptides to efficiently cross cell membranes and be cleaved into short peptides for presentation on MHC class I complexes [16]. To improve the immune response, MAGE-A3 has been tested with various immunostimulants, of which AS15 has produced the best clinical response in Phase II trials [17]. However, a Phase I dose escalation trial of MAGE-A3/HPV16 found that, despite good tolerability and the development of robust T cell and antibody responses, all 16 patients experienced disease progression [18]. Phase III trials of the MAGE-A3 vaccine in NSCLC and melanoma were also terminated prematurely due to futility, suggesting that MAGE-A3 vaccination is unlikely to be an optimal immunotherapeutic strategy [17,19]. Phase I trials testing MAGE-A3/HPV16 in HNSCC were scheduled for completion in 2012, however the current status and results from these trials is not known.

#### 3.2.2. Mucin-1

Mucin-1 (MUC1) is a heavily glycosylated protein that undergoes proteolytic cleavage to form heterodimeric complexes on the cell surface, where it helps to provide a protective barrier between the cell and the external environment [20]. Accordingly, MUC1 is expressed by most epithelial cells and is overexpressed in multiple tumor types. In tumor cells MUC1 promotes tumor growth, metastasis, and drug resistance, and the C-terminal tail may serve as an oncogenic signaling molecule [21]. Tumor-associated MUC1 is characterized by altered glycosylation patterns, which enable differential targeting of tumor MUC1 for vaccine therapy [22].

Indeed, in 2009 MUC1 was listed as the second-highest priority tumor antigen for vaccine therapy [23], and currently there are nearly 30 ongoing clinical trials involving MUC1, some of which are exploring the exciting possibility of using MUC1 vaccination for cancer prevention, in addition to cancer treatment. In general, MUC1 vaccines are well-tolerated, generate tumor-specific T cell responses, and have shown evidence of efficacy in a variety of tumor types. However, despite the large number of early-phase trials, only a few MUC1 vaccine programs have reached stage III clinical trials, and therefore the long-term utility of MUC1 vaccination for cancer treatment or prevention remains to be determined. A Phase I/II MUC1 vaccine trial for HNSCC is currently underway with a predicted completion date in 2021.

#### 3.2.3. AlloVax

The two-part AlloVax vaccine is comprised of chaperone protein-enriched tumor cell lysate from the patient’s own tumor and the AlloStim adjuvant, an intentionally mismatched allogenic stem cell transplant. This combination produces a host-versus-graft response called the “mirror effect” [24], which stimulates an eventual host-versus-tumor response. This approach has yielded T cell responses in vitro, however further clinical trials are required to determine the efficacy and safety profile of this vaccination strategy. Although AlloVax has not been subjected to extensive trials, Phase I and II trials are underway for HNSCC with estimated completion dates in 2016 and 2018, respectively.

#### 3.2.4. ISA101 and ISA201 (HESPECTA)

The ISA101 vaccine is a mixture of 13 overlapping 25–35 amino acid synthetic peptides derived from the HPV16 E6 and E7 proteins [25]. The peptide mixture (nine E6 peptides and four E7 peptides) is combined with the Montanide ISA-51 adjuvant, which contains all potential T cell epitopes and therefore produces T cell activation irrespective of the HLA type of the patient. Although results from trials of ISA101 in head and neck cancer patients have yet to be reported (Table 1), promising results have been observed in patients with HPV16-induced non-invasive vulvar and vaginal lesions. One trial of 20 patients with vulvar intraepithelial neoplasia demonstrated complete or partial response in 12 of 20 patients at 3 months and 15 of 19 patients at 12 months [26]. Furthermore, in a trial of 43 patients, HPV16-positive high-grade vulvar and vaginal intraepithelial neoplasia treated with ISA101 plus imiquimod or ISA101 alone, clinical response was observed at 3 months in 18 of 34 patients completing the vaccine series and in 15 of 29 patients evaluated at 12 months [27]. Imiquimod had no impact on response; however, in both trials clinical response was correlated with stronger vaccine-specific immune responses [26,27]. Trials of ISA101 for patients with HPV-positive head and neck cancers are currently underway.

ISA201 (HESPECTA) is a second generation vaccine based on ISA101 in which two HPV16 E6 peptides are conjugated to Amplivant, a synthetic Toll-like receptor-2 (TLR2) agonist [28]. A Phase I trial of HESPECTA in patients with non-metastatic HPV16-positive head and neck cancer is currently underway (Table 1). Results are expected in December of 2016.

### 3.3. Biologic Vaccines

#### 3.3.1. ADXS11-001

The ADXS11-001 vaccine consists of a live, attenuated strain of *Listeria monocytogenes* engineered to secrete the HPV-E7 protein fused to the *Listeria* listeriolysin O protein [29]. *Listeria monocytogenes* infects antigen-presenting cells, resulting in antigen presentation and activation of CD4+ and CD8+ T cells targeting HPV-infected tumor cells. A Phase I trial in 15 patients with metastatic, refractory, or recurrent cervical cancer treated with two vaccinations of 1 × 10^9^ colony forming units (CFUs), 3.3 × 10^9^ CFUs, or 1 × 10^10^ CFUs demonstrated median survival of 347 days, 1-year survival of 53%, and reduced tumor volume in 31% of patients. These results are particularly exciting when compared to historical controls, which have a median survival of 6 months and 1-year survival of 5% [29,30].

Similarly, preliminary results from the GOG-0265 trial demonstrated a 1-year survival rate of 38.5% for 26 women with persistent or recurrent metastatic cervical cancer treated with three doses of ADXS11-001 at 1 × 10^9^ CFU, compared to a 20% 1-year survival rate for historical controls [31]. In both trials, adverse events were manageable and primarily consisted of “flu-like” symptoms including fatigue, chills, fever, and headache. However, clinically-relevant hypotension has been reported, including dose-limiting diastolic hypotension that responded to intravenous (IV) fluid bolus in patients receiving 1 × 10^10^ CFU doses [29,31]. Phase II clinical trials to establish the efficacy of ADXS11-001 for HPV-positive oropharyngeal HNSCC are currently underway.

#### 3.3.2. Semi-Allogenic Human Fibroblast Vaccine

The theory underlying semi-allogenic cell transfer is that weakly immunogenic tumor-associated antigens can be converted to highly immunogenic antigens when presented by allogenic fibroblasts. To this end, fibroblast cell lines are transfected with DNA isolated from extirpated tumor cells, grown in culture, and then lethally irradiated before being injected into the patient from whom the tumor DNA was derived [32]. To our knowledge this approach has yet to be applied to human subjects, although studies in murine cancer models have produced promising results. In one study, LM murine fibroblast cells, genetically engineered to express interleukin-2 (IL-2), were transfected with genomic DNA isolated from a sporadic breast cancer in a C3H/He mouse before being injected into tumor-bearing C3H/He mice [33]. The mice that received fibroblast injections survived significantly longer than control animals, likely due to a robust CD8+ T cell response [33]. Similar approaches have been used in murine squamous cell carcinoma models [34], and the recipient fibroblast cell lines have been modified to optimize the immune response [35]. A current two-stage Phase I trial aims to expand this approach to human subjects with HNSCC, using the MRC-5 human fibroblast cell line [36]. MRC-5 cells will be transfected with genomic DNA isolated from extirpated HNSCC, grown in culture, and then lethally irradiated prior to being injected into the patient from whom the tumor DNA was isolated. Enrollment is anticipated to begin in 2016, with reporting of preliminary results by 2018.

## 4. Monoclonal Antibodies

**Figure 1:**
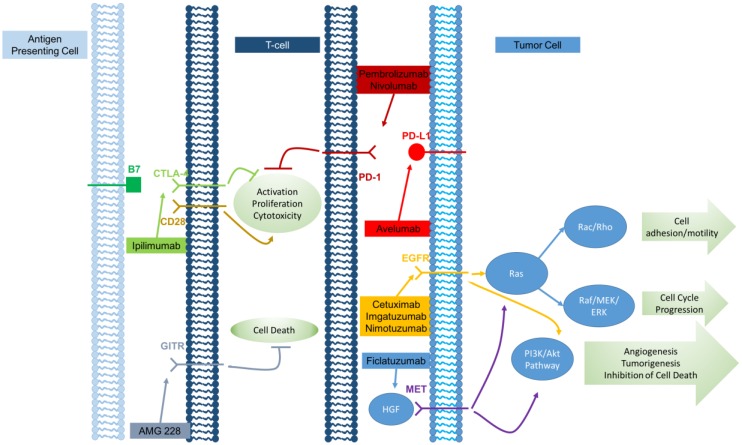
Immunomodulatory actions of monoclonal antibodies on head and neck squamous cell carcinoma. CTLA-4: cytotoxic T-lymphocyte-associated protein 4; GITR: glucocorticoid induced tumor necrosis factor superfamily member 18-related protein; PD-1: programmed cell death protein-1; PD-L1: programmed death-ligand 1; EGFR: epidermal growth factor receptor; MET: MET proto-oncogene, receptor tyrosine kinase; HGF: hepatocyte growth factor; PI3K/Akt: phosphoinositide-3 kinase/Akt pathway; ERK: extracellular signal-regulated kinase.

Over recent years, an increasingly diverse group of monoclonal antibodies have been applied to cancer therapy, representing a significant advance in cancer treatment. Although these antibodies target a range of molecules, many antibodies mediate their antitumor effects through similar mechanisms including the targeting of tumor cells for antibody-dependent cell-mediated cytotoxicity (ADCC), the direct inhibition of tumor growth signals, and inhibiting signaling pathways involved in maintaining immune self-tolerance. Figure 1 graphically depicts the various monoclonal antibodies and where they act along the immune pathway.

### 4.1. Receptor Tyrosine Kinase Inhibitors

#### 4.1.1. Cetuximab

Cetuximab is a humanized monoclonal murine antibody targeting the epidermal growth factor receptor (EGFR). EGFR is an erbB family receptor tyrosine kinase expressed by a wide variety of tumor types. Binding of a ligand to EGFR results in receptor dimerization, autophosphorylation, and induction of signaling cascades leading to cell proliferation. Cetuximab works by inhibiting ligand binding to EGFR, thereby blocking cell growth signaling [37]. Importantly, for colorectal cancer cetuximab is only effective for tumors expressing wild-type KRAS, as mutant KRAS provides growth signals that bypass EGFR inhibition. However, for HNSCC cetuximab appears to be effective for both KRAS-wild type and KRAS-mutant tumors [38]. Cetuximab was approved for HNSCC in 2006 after early Phase III trials demonstrated that cetuximab improved response to chemotherapy and reduced the risk of death for patients who have cetuximab-related skin toxicity [39].

Later studies showed that cetuximab plus platinum-based chemotherapy increases overall and progression-free survival (PFS), and that 5-year survival is improved for patients receiving chemoradiotherapy who developed skin rash of grade ≥2 severity in response to cetuximab [40,41]. However, a more recent Phase III study of >800 patients found that adding cetuximab to platinum-based chemoradiotherapy regimens did not improve PFS or OS, suggesting that routine cetuximab administration may not be beneficial to all patients [42]. Therefore, further research is needed to identify patients who are most likely to benefit from cetuximab therapy. Currently, there are over 80 ongoing clinical trials examining the use of cetuximab for various cancers, including four trials for HNSCC.

#### 4.1.2. Imgatuzumab (GA201)

Imgatuzumab is a first-in-class glycoengineered humanized anti-EGFR monoclonal antibody. The unique characteristics of imgatuzumab result from the addition of bisected afucosylated carbohydrate moieties to the Fc domain, which enhances the interaction with Fcγ receptors on immune effector cells and produces enhanced antibody-dependent cell-mediated cytotoxicity [43]. Phase I/II trials in patients with colorectal carcinoma demonstrated an acceptable safety profile, with skin rash as the most common adverse effect. Moreover, therapeutic responses were observed in patients with KRAS-mutant tumors, indicating that imgatuzumab may be more beneficial than cetuximab for this patient population [44,45]. In addition, one trial of 25 patients on single-agent imgatuzumab therapy produced a median OS of 9.3 months, which was longer than previously reported OS of 4.5 months for single-agent cetuximab therapy [45]. Although further data are required, these results, as well as preclinical data from mouse model systems, suggest that imgatuzumab may be more efficacious for the treatment of solid tumor than existing anti-EGFR therapies [43]. A Phase I trial of imgatuzumab was started in 2009; however, the drug testing pipeline was cancelled in 2013 before results were released.

#### 4.1.3. Nimotuzumab

Nimotuzumab is a humanized anti-EGFR monoclonal antibody notable for its unique safety profile, where antitumor activity has been observed in the absence of the severe skin, mucosal, renal, and gastrointestinal toxicity observed with cetuximab [46,47]. A phase IIb trial in 92 Indian patients with stage III or IV HNSCC treated with nimotuzumab plus chemoradiation therapy versus single modality radiation therapy demonstrated 30-month PFS of 57% in the nimotuzumab group versus 22% in the control group [47]. Moreover, while median OS was not achieved by 30 months for the nimotuzumab group, OS was 22 months for the control group [47]. A similar phase IIb study of 76 Indian patients comparing nimotuzumab plus chemoradiation therapy, nimotuzumab plus radiation therapy, chemoradiation therapy, and radiation therapy alone demonstrated improved survival in patients receiving nimotuzumab, with maximal OS in the nimotuzumab plus chemoradiation therapy group [48]. While further evidence is required, these data together suggest that nimotuzumab is a promising therapeutic agent for HNSCC. A Phase III trial of nimotuzumab is currently recruiting HNSCC patients, with results expected in 2021.

#### 4.1.4. Ficlatuzumab

In contrast to the previously discussed monoclonal antibodies, which all target EGFR, ficlatuzumab is a humanized monoclonal antibody targeting the hepatocyte growth factor (HGF). HGF is the only known ligand of the cellular MET tyrosine kinase receptor, which activates cellular signaling cascades including PI3K/Akt, Ras/Rac/Rho, and Ras/MAPK, leading to cancer growth, metastasis, angiogenesis, and drug resistance [49]. Importantly, overexpression of HGF and cMET has been identified in HNSCC, suggesting that immunotherapies targeting this pathway may be beneficial for this patient population [49,50]. Preclinical studies of ficlatuzumab monotherapy for non-small cell lung cancer (NSCLC), which has a similar genetic profile to HNSCC [51], resulted in reduced tumor growth and decreased levels of phospho-MET, phospho-ERK, and phospho-Akt, but a concurrent increase in phospho-EGFR.

A combination of ficlatuzumab plus cetuximab therapy resulted in complete tumor response in all animals, indicating a role for dual inhibition of the HGF and EGFR pathways [52]. Unfortunately, a phase II trial of ficlatuzumab plus the EGFR inhibitor gefitinib versus gefitinib alone showed no difference in PFS, although biomarker analysis demonstrated a paradoxically increased response to ficlatuzumab therapy for patients with activating EGFR mutations and lower cMET expression [53]. Therefore, ficlatuzumab may be best suited for highly-selected patients with specific mutational and gene expression profiles. Phase I trials for ficlatuzumab in HNSCC patients are currently underway, with projected completion dates in 2018.

### 4.2. Checkpoint Inhibitors

Immune checkpoints limit the inflammatory response, reducing damage to normal tissues and preventing autoimmunity. Therapies that inhibit or disable these checkpoints may therefore break immune tolerance to TAAs and induce a host antitumor immune response. Currently, five checkpoint inhibitors are commercially available or are undergoing clinical trials. Three checkpoint inhibitors target programmed death-1 (PD-1) pathway. PD-1 is a cell surface receptor expressed on activated B and T cells that binds the PD-L1 and PD-L2 ligands. Because PD-1 activation by ligand binding decreases the inflammatory response [54], inhibiting PD-1 by direct antibody binding or by targeting PD-L1 or PD-L2 may relieve immune tolerance, promoting immune cell-mediated tumor lysis. Other target pathways include CTLA-4, which is expressed on the surface of activated T cells and limits T cell activity, and the glucocorticoid induced tumor necrosis factor receptor (GITR), a T cell costimulatory molecule. Importantly, effective checkpoint inhibition therapy requires the presence of inhibited CD8+ T cells at the tumor periphery [55].

#### 4.2.1. Pembrolizumab

Pembrolizumab is a humanized monoclonal antibody targeting PD-1 that is currently Food and Drug Administration (FDA) approved as monotherapy for metastatic melanoma and metastatic NSCLC. In addition, pembrolizumab has shown promise for HNSCC. Specifically, preliminary results from the KEYNOTE-012 trial, which was comprised of patients with recurrent HNSCC, demonstrated an overall response rate of 18.2% and a disease control rate of nearly 50% [56,57]. Furthermore, pembrolizumab was better tolerated than aggressive chemoradiation therapy, with the most common adverse effects being fatigue, hypothyroidism, decreased appetite, and rash [57]. Survival will be assessed as part of ongoing phase II and III trials; however, these early results suggest that PD-1 inhibition may be an effective target for HNSCC.

#### 4.2.2. Nivolumab

Similar to pembrolizumab, nivolumab is a humanized monoclonal anti-PD-1 antibody that is FDA approved for the treatment of renal cell carcinoma, NSCLC, and melanoma. For HNSCC, the phase III CheckMate-141 trial, which compared nivolumab to investigators’ choice of cetuximab, docetaxel, or methotrexate, was terminated early due to superiority of the nivolumab arm over the control arm [58]. Although results from CheckMate-141 have yet to be published, studies in NSCLC have demonstrated a clear survival benefit for nivolumab versus docetaxel chemotherapy, particularly among patients whose tumors expressed PD-1L [59]. Therefore, these data provide additional evidence that immune checkpoint inhibition may be a valuable therapeutic approach for HNSCC.

#### 4.2.3. Avelumab

In contrast to many of the monoclonal antibodies discussed to this point, avelumab is a fully human monoclonal anti-PD-L1 antibody. Importantly, preclinical studies have indicated that avelumab is capable of inducing antibody-dependent cell-mediated cytotoxicity [60,61], which may play a critical role in cancer immunotherapy [62]. An open-label phase Ib trial of avelumab in patients with NSCLC that progressed on platinum therapy demonstrated complete or partial response in 12% of patients and stable disease in an additional 38% [63]. Although early trials have indicated that adverse effects are common with avelumab, the drug appears to have an acceptable safety profile, with the most common adverse effects being fatigue, nausea, infusion reaction, diarrhea, chills, decreased appetite, pyrexia, flu-like illness, and arthralgia [63,64]. While early results are promising, the ultimate utility of avelumab for HNSCC will be determined by ongoing phase I, II, and III trials.

#### 4.2.4. Ipilimumab

Ipilimumab differs from the checkpoint inhibitors discussed thus far in that this humanized monoclonal antibody targets CTLA-4, an immunomodulatory protein expressed on the surface of activated T cells. CTLA-4 binds to B7, which prevents B7 from interacting with the co-stimulatory molecule CD28, limiting T cell proliferation and IL-2 production [65]. Blocking CTLA-4 relieves this T cell inhibition, resulting in a host antitumor immune response. Phase III trials of ipilimumab in patients with metastatic melanoma demonstrated improved OS for patients receiving ipilimumab [66], which lead to FDA approval of the drug for unresectable or metastatic melanoma in 2011. Unfortunately, trials of ipilimumab have produced less promising results for lung cancer. It is important to note that ipilimumab exhibits unique tumor response kinetics including response after initial progression and objective responses occurring up to 6–12 months following treatment [65].

A large Phase II study of patients with NSCLC or small cell lung cancer (SCLC) found that while ipilimumab increased overall PFS and immune-related PFS, which accounts for the unique response characteristics of ipilimumab [67], there was no increase in OS for either SCLC or NSCLC patients [68,69]. Additionally, the greatest increase in PFS was observed among patients who received ipilimumab after chemotherapy, indicating that ipilimumab is most efficacious following tumor antigen release [68,69]. Importantly, ipilimumab produces a unique side-effect profile due to immune system activation, including diarrhea, enterocolitis, intestinal perforation, and hepatotoxicity [70]. Taken together, these data suggest that ipilimumab may have promise for HNSCC, but investigators should be aware of the unique characteristics of this medication when considering any patient for this therapy. Ipilimumab is currently undergoing a Phase I/II trial for virus-associated tumors, including HNSCC, with a projected completion date in 2018.

#### 4.2.5. AMG 228

AMG 228 is a novel monoclonal antibody currently undergoing Phase I safety trials for a variety of solid tumors including HNSCC. This antibody targets GITR, which is expressed on the surface of CD25+ CD4+ regulatory T cells and acts as an effector T cell co-stimulatory molecule, possibly by inhibiting T cell death [71]. In animal model systems, GITR stimulation results in decreased self-tolerance and the development of autoimmunity [72]. In addition, administration of GITR agonist antibodies in murine melanoma model systems results in tumor immunity and rejection [73,74]. Accordingly, GITR modulation has been listed as one of the National Cancer Institute’s top 25 most promising research areas [71]. Results from the initial human trials of AMG 228 in solid tumors are expected in 2017.

## 5. Oncolytic Viruses and Active Immunotherapeutics

Oncolytic viruses represent a novel approach to cancer therapy that uses recombinant or engineered viruses to selectively kill tumor cells while sparing normal tissues. In addition, tumor cell lysis releases TAAs into the surrounding environment along with viral antigens, which may facilitate the production of an immune antitumor response. Currently, two oncolytic viruses are undergoing clinical trials for HNSCC.

### 5.1. Pexa-Vec (JX-594)

Pexa-Vec is a recombinant vaccinia virus (family *Poxviridae*) that is deleted for the viral thymidine kinase (vTK) gene and contains an exogenous Granulocyte Macrophage – Colony Stimulating Factor (GM-CSF) gene [75]. The vTK deletion renders the virus replication-deficient in normal human cells that express constitutive levels of cellular thymidine kinase (cTK), but permits virus replication in tumor cells that overexpress cTK [76,77]. In addition, Pexa-Vec replication is stimulated by EGFR/Ras signaling and type-I interferon resistance, giving the virus both relative selectivity for tumor cells and the ability to replicate in tumors with diverse genetic alterations [77]. Because vaccinia, like all poxviruses, is highly cytolytic, viral replication results in direct oncolysis in addition to stimulation of GM-CSF sensitive leukocytes, such as neutrophils [75]. The virus was originally developed for use in hepatocellular carcinoma, where Phase II trials have demonstrated dose-related oncolysis, antitumor immunity, and increased OS with direct intratumor injection [78]. Despite the administration of Pexa-Vec via intratumor injections in initial trials, the virus is stable for intravenous administration although relatively high doses may be required to achieve a systemic antitumor effect [79]. Systemic Pexa-Vec administration has an acceptable safety profile with mild adverse effects including pyrexia, chills, headache, nausea, vomiting, and grade I papulopustular rash [79]. Accordingly, Pexa-Vec may be an exciting option for the treatment of a variety of solid tumors including HNSCC. A Phase I trial of Pexa-Vec in HNSCC has been completed, although the results have not yet been published.

### 5.2. TRICOM

TRICOM is a recombinant avian fowlpox virus that infects mammalian cells but is replication-deficient in mammals [80]. The recombinant virus used in fowlpox-TRICOM expresses three costimulatory transgenes, B7.1 (CD80), Intercellular Adhesion Molecule 1 (ICAM-1), and lymphocyte function-associated antigen 3 (LFA-3), plus one or more tumor-associated antigens, such as Carcinoembryonic antigen (CEA) and MUC-1 [80]. In a Phase II trial where patients with metastatic colorectal carcinoma received dendritic cells infected with CEA/MUC-1 TRICOM (PANVAC), OS was not reached by 72 months of follow-up for patients receiving PANVAC, but was 44 months in contemporary controls, indicating that PANVAC therapy may improve survival for patients with solid tumors. In addition, pilot studies of systemic PANVAC therapy for patients with metastatic ovarian or breast carcinoma have suggested that this approach may provide clinical benefit to selected patients [81]. The vaccine appears to be well-tolerated and the virus itself elicits only a limited immune response, allowing the virus to be administered repeatedly to achieve a more robust antitumor immune response [80,81]. Phase I studies of the PANVAC vaccine in head and neck cancer patients are nearing completion.

## 6. Immunomodulators

For the purposes of this review, we define immunomodulators as a diverse group of small molecule agonists and compounds designed to increase the host antitumor immune response. In general, these compounds result in generalized immune system stimulation, rather than a directed antitumor response.

### 6.1. Toll-Like Receptor Agonists

TLRs are a family of transmembrane signaling proteins that form an important component of the innate inflammatory response by detecting conserved microbe-associated molecular patterns (MAMPs) and endogenous products known as damage-associated molecular patterns (DAMPs) [82]. Accumulating evidence indicates that TLR expression is altered in malignant cells and that DAMP release by dying cancer cells results in TLR activation, which may contribute to clinically relevant antitumor inflammatory responses [82,83]. Based on these observations, several TLR agonists have been developed for cancer immunotherapy applications.

#### 6.1.1. Motolimod (VTX-2237)

Motolimod is a small molecule TLR8 agonist that enhances monocyte, dendritic cell, and natural killer (NK) cell activation and increased ADCC [84]. Pre-clinical studies have indicated that motolimod stimulation of peripheral blood mononuclear cells (PBMCs) isolated from healthy donors increases cetuximab-mediated lysis of cultured HNSCC cells [85]. Phase I trials have demonstrated that motolimod has an acceptable safety profile and increases NK cell activation in HNSCC patients, which may augment NK-mediated tumor lysis in response to other therapies, such as cetuximab [86,87]. However, further studies are needed to demonstrate the overall efficacy of motolimod for HNSCC patients. Results from a Phase Ib trial of motolimod in HNSCC are expected this year.

#### 6.1.2. IMO-2055 (EMD 1201081)

Similar to motolimod, IMO-2055 is a synthetic TLR9 agonist that enhanced NK cell activation and stimulated a potent antitumor immune response in combination with monoclonal antibody therapy in preclinical trials [88,89]. However, a phase I study of IMO-2055 provided only limited evidence that IMO-2055 produces clinically-relevant antitumor effects in vivo [90]. A further Phase II study of IMO-2055 plus cetuximab versus cetuximab monotherapy for HNSCC patients also failed to demonstrate clinical efficacy [91], limiting potential applications of IMO-2055 for HNSCC. Importantly, evidence from other Phase I and III trials has indicated that IMO-2055 and other TLR9 agonists increase known myelosuppressive effects of platinum-based chemotherapy, which may lead to serious and potentially fatal adverse effects including hypotension, febrile neutropenia, and septicemia [92,93,94]. Together, these data indicate that IMO-2055 likely has an unacceptable risk to efficacy ratio and may not be appropriate for clinical use. A Phase I study of IMO-2055 in HNSCC was terminated before results were released.

#### 6.1.3. Picibanil (OK-432)

In contrast to the synthetic TLR agonists discussed above, picibanil is a lyophilized preparation of the *Streptococcus pyogenes* su-strain that has been treated with benzylpenicillin [95]. Originally developed and approved for the treatment of lymphangiomas [96,97], picibanil induces a substantial immune inflammatory response, likely via TLR4 signaling pathways [95]. Notably, picibanil has been in clinical use for over 30 years, and therefore has a well-characterized safety and adverse effect profile. Strikingly, pre-clinical studies have indicated that active components of picibanil may induce direct lysis of head and neck cancer cells, suggesting that picibanil may induce HNSCC regression both by direct and immunostimulatory effects [98]. Indeed, a Japanese study of 81 patients with oral squamous cell carcinoma who were treated with picibanil plus chemoradiotherapy versus chemoradiotherapy alone found significantly increased OS and PFS among patients who received picibanil [99]. Accordingly, picibanil may be a useful adjuvant to standard therapeutic approaches for head and neck cancers. A Phase I trial of picibanil was completed in 2012, however results have not been published.

### 6.2. Exogenous Cytokines

Cytokines are a diverse family of small, non-structural signaling proteins that play diverse roles in modifying immune and inflammatory responses [100]. Importantly, increasing evidence indicates that tumors modulate the local cytokine milieu to produce a pro-inflammatory environment while simultaneously facilitating tumor immune system evasion [101]. Therefore, altering the cytokine response by providing exogenous cytokine mixtures may facilitate the immune antitumor response.

#### 6.2.1. IL-12

One mechanism by which tumors evade immune recognition is through local immunosuppression, resulting in downregulation of the IL-12–interferon γ–HLA-DR axis [101]. Therefore, increasing local IL-12 levels via an exogenous plasmid vector may facilitate tumor clearance by the immune system. In animal model systems, intratumoral electroporation of an IL-12 expression plasmid (pIL-12) resulted in significant intratumoral IL-12 expression, leading to disease stabilization and eventually complete regression without systemic toxicity [102]. Phase I trials of intratumoral plasmid IL-12 electroporation in patients with metastatic melanoma resulted in a dose-dependent increase in tumor cell necrosis and lymphocyte infiltration, in addition to stabilization or regression of non-electroporated metastases in over 50% of treated patients [103]. As in animal systems, intratumoral electroporation was associated with minimal systemic effects [103], indicating that plasmid IL-12 preparations may be a promising therapy for advanced or metastatic disease. Current trials are examining the efficacy of IL-12 delivered as liposomal or plasmid preparations for HNSCC.

#### 6.2.2. IRX-2

IRX-2 is a biologic immunotherapeutic containing a mixture of cytokines including IL-1β, IL-2, IL-6, IL-8, TNFα, GM-CSF, and IFN-γ [104]. Preclinical studies have shown that this cytokine cocktail is active on multiple immune cell types, inducing dendritic cell maturation, T cell activation, and NK cell stimulation [104,105,106,107]. Phase I and II trials have demonstrated that IRX-2 is safe and generally well-tolerated, although some patients may develop potentially serious adverse effects including anemia and lymphopenia [108,109]. In addition, a Phase II study of 42 head and neck cancer patients treated with IRX-2 plus chemoradiotherapy demonstrated higher OS and longer time to recurrence compared to contemporary off-study controls [108]. IRX-2 treatment was associated with symptomatic improvement in 57% of patients [108], suggesting that IRX-2 may be an efficacious treatment to augment traditional chemoradiotherapy and surgery for head and neck cancers. A phase II trial examining the use of IRX-2 for HNSCC patients is currently underway, with an estimated completion in 2019.

## 7. Future Directions

One exciting potential application of immunotherapeutics, particularly vaccine therapies, is cancer prevention by vaccinating selected high-risk patients against TAAs. In the case of HPV-associated cancer, vaccination against high-risk HPV strains results in a clear reduction in the incidence of cervical cancer [110], and has the potential to prevent >90% of HPV-positive head and neck cancers [111]. However, the effect of HPV vaccination after the establishment of persistent HPV is not known. Moreover, it remains to be determined whether vaccination against HPV antigens, such as E6 or E7, will be an effective cancer prevention strategy for individuals with persistent HPV infection at high risk for developing HPV-associated oropharyngeal cancer. Identifying individuals with persistent oropharyngeal HPV infection also remains a barrier to cancer preventative strategies, as there is currently no standardized test to identify HPV-associated premalignant oropharyngeal lesions.

Another area of HNSCC immunotherapeutics requiring further study is the ability to identify, *a priori*, which patients are likely to benefit from a particular immunotherapeutic approach. Indeed, for many immunotherapeutics only a subset of patients respond to any given therapy, likely due to variations in HLA subtype or other immunomodulatory proteins. In addition, the specific mutational and gene expression profile of a given tumor may also have significant impacts on the effectiveness of immunotherapy. For some immunotherapeutics, such as the Allovectin-7 vaccine, there are described molecular predictors of immune response, however for a vast majority of therapies the specific molecular and genetic characteristics that predict response remain to be defined. Therefore, integrated genomic characterization of both germline and tumor tissues from HNSCC patients may lead to the identification of molecular profiles that predict responses to a given therapy. However, it should be noted that due to the divergent mechanisms of action among many immunotherapeutics, molecular profiles that predict response to one agent may not be broadly predictive for general responses to immunotherapies.

## 8. Conclusions

Immunotherapy represents a promising avenue for the treatment of head and neck cancers, with several treatment regimens showing significant promise in clinical trials. When combined with traditional approaches including chemotherapy, radiation therapy, and surgery, these immunotherapies have the potential to reduce the morbidity associated with HNSCC and improve survival. It should be noted, however, that immunotherapies often produce a response profile that is often significantly different from that associated with traditional chemotherapy. Indeed, many immunotherapeutics require a longer period of time to achieve clinical response, and may even induce tumor pseudo-progression, when compared to traditional chemotherapeutic approaches. Therefore, these agents may not be the modality of choice in cases where the tumor is endangering critical structures or there is impending airway compromise. Thus, initial surgical debulking may still play an important role for HNSCC patients treated with immunotherapy. Importantly, one particularly exciting feature of immunotherapy may be longer-term responses compared to other modalities, although further data are required to determine whether immunotherapy, particularly vaccine therapies, can produce durable responses.

## Figures and Tables

**Table 1:** Current immunotherapy clinical trials for head and neck squamous cell carcinoma.

Therapy	Immune Target	Stage	Number Enrolled	Clinical Trial Number(s)
Vaccine Therapies				
INO-3112	HPV E6, E7	Phase I/II	25	NCT02163057
Allovectin-7^®^	Tumor antigens via MHC-I expression	Phase II/III	Not Reported	NCT00050388
MAGE-A3/HPV16	MAGE-A3, HPV-16-specific peptide	Phase I Phase I	90 48	NCT00257738 NCT00704041
MUC1 Vaccine	MUC1	Phase I/II	104	NCT02544880
AlloVax	Chaperone-enriched tumor cell lysate	Phase II Phase I/II	100 52	NCT02624999 NCT01998542
ISA101	Synthetic HPV E6 and E7 peptides	Phase II	28	NCT02426892
HESPECTA (ISA201)	Two synthetic HPV16 peptides covalently linked to AMPLIVANT^®^ synthetic TLR 1/2 ligand	Phase I	24	NCT02821494
ADXS11-001	Live, attenuated *Listeria monocytogenes* expressing HPV-E7-lysteriolysin-O fusion	Phase II	30	NCT02002182
Semi-allogenic human fibroblasts	Patient-derived tumor-associated antigens	Phase I	37	NCT02211027
Monoclonal Antibodies				
Cetuximab	EGFR	Phase II Phase I Phase II Phase I	40 24 114 22	NCT01218048 NCT02124850 NCT02707588 NCT02277197
Imgatuzumab (GA201, RO5083945)	EGFR	Phase I	62	NCT01046266
Nimotuzumab	EGFR	Phase III	710	NCT00957086
Ficlatuzumab	Hepatocyte growth factor	Phase I Phase I	22 24	NCT02277197 NCT02277184
Pembrolizumab (MK-3475)	PD-1	Phase II Phase I/II Phase II Phase I/II Phase III	46 400 114 22 780	NCT02296684 NCT02452424 NCT02707588 NCT02718820 NCT02358031
Nivolumab	PD-1	Phase I Phase II Phase I/II Phase II	24 40 199 28	NCT02124850 NCT02684253 NCT02488759 NCT02426892
Avelumab	PD-L1	Phase I	1670	NCT01772004
Ipilimumab	CTLA-4	Phase I/II	199	NCT02488759
AMG 228	GITR	Phase I	100	NCT02437916
Oncolytic Viruses and Active Immunotherapeutics				
Pexa-Vec	Recombinant vaccinia virus, deleted for viral thymidine kinase and expressing GM-CSF	Phase I	23	NCT00625456
TRICOM	Recombinant fowlpox virus expressing B7.1, ICAM-1, LFA-3, CEA, MUC-1	Phase I	Not Reported	NCT00021424
Immunomodulators				
Motolimod	TLR8 agonist	Phase I Phase I	24 13	NCT02124850 NCT01334177
Picibanil (OK-432)	Immunostimulant via TLR4 pathway	Phase I	10	NCT01149902
IL-12	Proinflammatory cytokine	Phase II Phase I/II Phase II	31 34 Not Reported	NCT02345330 NCT00004070 NCT00006033
IRX-2	Cytokine mixture: IL-1β, IL-2, IL-6, IL-8, TNFα, GM-CSF, IFN-γ	Phase II	400	NCT02609386

HPV: Human Papilloma Virus; MHC-I: Major Histocompatibility Complex Type I; MAGE-A3: Melanoma-associated Antigen 3; MUC1: Mucin-1; TLR: Toll-like Receptor; EGFR: Epidermal Growth Factor Receptor; PD-1: Programmed cell death protein-1; PD-L1: Programmed death-ligand 1; CTLA-4: Cytotoxic T-lymphocyte-associated protein 4; GITR: Glucocorticoid Induced Tumor Necrosis Factor superfamily member 18-related protein; GM-CSF: Granulocyte Macrophage–Colony Stimulating Factor; ICAM-1: Intercellular Adhesion Molecule 1; LFA-3: lymphocyte function-associated antigen 3; CEA: Carcinoembryonic antigen; IL: interleukin; TNFα: Tumor Necrosis Factor-alpha; IFN-γ: Interferon-gamma.
